# Digital Pathology: How Far Are We from Automated Tissue-Based Diagnosis?

**DOI:** 10.1155/2014/458954

**Published:** 2014-12-21

**Authors:** Klaus Kayser, Stephan Borkenfeld, Amina Djenouni, Joachim Christian Manning, Herbert Kaltner, Gian Kayser, Hans-Joachim Gabius

**Affiliations:** ^1^Charité-Universitätsmedizin Berlin, Berlin, Germany; ^2^IAT Heidelberg, Heidelberg, Germany; ^3^Pathology Institute, Batna, Algeria; ^4^Maximilians University, Munich, Germany; ^5^Institute of Pathology, University of Freiburg, Freiburg, Germany

## Background

Tissue based diagnosis (TBD) includes all diagnostic procedures that are performed on human tissue for disease classification and treatment. Its computerized information analysis is called Digital Pathology. Herein we will discuss the present stage of IT tools and the assumed clinical perspectives on medical performance and treatment.

## Theory

Basically, TBD investigates the function and structures of biological meaningful individual units, such as macromolecules, genes, nuclei, cells, vessels, and organs. All functions are bound to structures that ensure reliable and effective information and energy exchange. Disturbance of structures induces less effective or complete loss of functions. The complex interactions at molecular biological level (macromolecules) and their continuous reproduction require extensive computations in addition to the sophisticated biochemical analysis systems. Nearly all assessable information is of visual nature or can be visualized. Thus, image content analysis applied in a sophisticated manner might be one key procedure to assist human image interpretation or to even replace it.

Image content information includes information that can be derived from predefined functional units (objects), their spatial arrangement (structure), pixel derived features prior of after image transformations (texture), and syntactic compositions of objects or of pixel based primitives (syntactic structure analysis). Statistically significant clusters can represent either new biological significant units (e.g., tubular arrangement of specific (endothelial) cells forming a vessel, spatial composition of cells of different nature (cellular sociology) forming a bronchus with assumed participation of endogenous lectins [1]) or other new items such as entropy flow charts and diffusion densities. All these parameters form a powerful set of image information features. They can be considered to be independent from each other and calculated independently for their specific clinical significance (disease association).

## Present Status

The development of whole slide image scanners, their implementation into laboratory and hospital information systems, and development of internet based open access image measurement systems permit the construction of automated disease classifiers with inbuilt image quality control and monitoring. The prerequisites include image standardization (of gray value range, distribution, magnification in relation to object measurements, etc.), detection of regions of interest (ROI), standardized image transformation procedures, and sophisticated statistical algorithms of flexible and self-learning classifiers.

## Perspectives

No technical constraint seems to exist that would prohibit a self learning automated disease detection system based upon TBD. Individual bricks are already available, and have been tested in still crude but promising trials. Internet test forums such as EAMUSTM or the recently created Virtual International Pathology Institute (VIPI, http://www.diagnomx.eu/vipi/) can be considered to be milestones in developing IT support in TBD and surgical pathology. The pathologist and the medical community can decide about velocity and support, not about <yes> or <no>.
